# New Nanostructured Carbon Coating Inhibits Bacterial Growth, but Does Not Influence on Animal Cells

**DOI:** 10.3390/nano10112130

**Published:** 2020-10-27

**Authors:** Eduard M. Barkhudarov, Igor A. Kossyi, Andrey M. Anpilov, Petr I. Ivashkin, Konstantin V. Artem’ev, Igor V. Moryakov, Mamikon A. Misakyan, Nick Christofi, Dmitry E. Burmistrov, Veronika V. Smirnova, Veronika V. Ivanyuk, Nikolay F. Bunkin, Valery A. Kozlov, Nikita V. Penkov, Mars G. Sharapov, Mikhail Yu. Volkov, Mikhail A. Sevostyanov, Andrey B. Lisitsyn, Anastasia A. Semenova, Maksim B. Rebezov, Sergey V. Gudkov

**Affiliations:** 1 Prokhorov General Physics Institute of the Russian Academy of Sciences, 119991 Moscow, Russia; barkhudarov@yandex.ru (E.M.B.); kossyi@fpl.gpi.ru (I.A.K.); anpilov@kapella.gpi.ru (A.M.A.); ivashkin@kapella.gpi.ru (P.I.I.); artemievkv@mail.ru (K.V.A.); moryakov@fpl.gpi.ru (I.V.M.); misakyan@fpl.gpi.ru (M.A.M.); dmitriiburmistroff@gmail.com (D.E.B.); veronausckova@mail.ru (V.V.S.); veronika.ivaniuk@yandex.ru (V.V.I.); nbunkin@mail.ru (N.F.B.); v.kozlov@hotmail.com (V.A.K.); rebezov@yandex.ru (M.B.R.); 2 Higher School of Economics, National Research University, 101000 Moscow, Russia; 3 School of Applied Sciences, Edinburgh Napier University, Edinburgh EH11 4DY, UK; n.christofi@napier.ac.uk; 4 Faculty of Fundamental Sciences, Bauman Moscow State Technical University, 105005 Moscow, Russia; 5 Federal Research Center “Pushchino Scientific Center for Biological Research of the Russian Academy of Sciences”, Institute of Cell Biophysics of the Russian Academy of Sciences, 142290 Pushchino, Russia; nvpenkov@yandex.ru (N.V.P.); sharapov.mars@gmail.com (M.G.S.); 6 Institute of Information Technologies, MIREA—Russian Technological University, 119454 Moscow, Russia; volkov_m@mirea.ru; 7 A.A. Baikov Institute of Metallurgy and Materials Science of the Russian Academy of Sciences, 119334 Moscow, Russia; cmakp@mail.ru; 8 V.M. Gorbatov Federal Research Center for Food Systems of Russian Academy of Sciences, 109316 Moscow, Russia; info@fncps.ru (A.B.L.); semmm@mail.ru (A.A.S.); 9 All-Russian Research Institute of Phytopatology, 143050 Bol’shie Vyazemy, Russia; 10 The Institute of Biology and Biomedicine, Lobachevsky State University of Nizhni Novgorod, 603950 Nizhni Novgorod, Russia

**Keywords:** nanosized carbon, antibacterial coatings, cytocompatibility

## Abstract

An electrospark technology has been developed for obtaining a colloidal solution containing nanosized amorphous carbon. The advantages of the technology are its low cost and high performance. The colloidal solution of nanosized carbon is highly stable. The coatings on its basis are nanostructured. They are characterized by high adhesion and hydrophobicity. It was found that the propagation of microorganisms on nanosized carbon coatings is significantly hindered. At the same time, eukaryotic animal cells grow and develop on nanosized carbon coatings, as well as on the nitinol medical alloy. The use of a colloidal solution as available, cheap and non-toxic nanomaterial for the creation of antibacterial coatings to prevent biofilm formation seems to be very promising for modern medicine, pharmaceutical and food industries.

## 1. Introduction

In materials science, a coating is a thin layer of another material applied to the surface of an object. The main purpose of coating is to improve the surface properties of the substrate. Almost any property may be improved by coatings. For example, resistance (to fracture, to high temperatures, to corrosion, etc.), adhesion, wettability, electrical conductivity, etc. In biomedicine, coatings improve the biocompatibility of implantable materials [1]. Conversely, in the food industry, coatings are used to provide materials with bactericidal or bacteriostatic properties [2]. Coatings may be applied as liquids, gases or solids, but as a result they are integral to substrate. In recent decades, coatings based on various allotropic forms of carbon (fullerenes, carbon nanotubes, graphene-like coatings, diamond-like coatings, etc.) are popular among researchers. The advantages of coatings based on various allotropic forms of carbon are in their extremely different physical and chemical properties and in the ability to form micro- and nanostructured surfaces [3]. Thus, fullerenes and carbon nanotubes are mainly used to achieve local effects in the periimplant tissues, since these materials have various biological effects. Fullerene derivatives were shown to exhibit antimicrobial properties against a wide range of microorganisms [4]. Surface-modified multi-walled carbon nanotubes (MWCNTs) were found to inhibit the activity of pathogenic microorganisms such as Pseudomonas aeruginosa and Staphylococcus aureus [5]. A number of C_60_ carboxyfullerene derivatives exhibit antiviral activity [6]. It was shown that C_60_ fullerenes protect the mammalian body from oxidative stress and are excellent antioxidants [7]. Glycofullerenes inhibit inflammatory responses, including those caused by particulate matter [8]. Fullerenols C_60_OH_24_ exhibit cardioprotective effects by decreasing the cardiotoxicity of doxorubicin in rats [9]. MWCNTs are considered potentially effective against Alzheimer’s disease [10]. This is likely due to the ability of MWCNTs to inhibit Aβ42 fibrillogenesis, as well as to disaggregate mature fibrils, which protects the body from cytotoxicity induced by Aβ42 [11]. It should be noted that fullerenes and carbon nanotubes are actively used not only for the treatment of neurodegenerative diseases, but also for their diagnostics [12]. In general, fullerenes and nanotubes, depending on the chemical modification, may exhibit abiotic properties and help in the treatment of various diseases. We have not found any references in the literature to derivatives of fullerenes or carbon nanotubes, which are both biocompatible for animal cells and exhibit antibacterial properties.

Graphene-like coatings are used for an insulating effect, mainly in reducing the risk of negative surface effects and release of potentially toxic compounds from the implant. In addition, graphene derivatives exhibit significant antimicrobial properties. It is assumed that the antimicrobial effect of graphene is related to the number of its layers [13]. With an increase in the number of graphene layers, its thickness increases. An increase in thickness probably leads to a weakening of the “nanoknife” effect [14]. In terms of energy, it is theoretically predicted that three-layer graphene may more easily penetrate a lipid bilayer compared to single-layer graphene [15]. This means that multilayer graphene has a higher ability to damage biological membranes and kill cells compared to single-layer graphene [16].

Diamond-like carbon (DLC) coatings are potential materials for use in orthopedics and traumatology. The main advantages of DLC are high hardness, chemical inertness, low friction factor, acceptable corrosion and wear resistance [17]. The absence of acute toxic effects of DLC coatings has been proven using cell cultures [18]. Experiments with animals have shown that DLC coatings do not induce an inflammatory response [19]. DLC coatings also have no significant effect on bacterial cells [20].

In general, carbon-based coatings have many benefits. This study presents a simple plasma technology for obtaining a colloidal solution of amorphous carbon nanoparticles, which may be applied to surfaces using simple devices such as a spray gun and even a brush. After drying on the surface, the colloidal solution forms a durable structured coating. The coating has bacteriostatic properties but does not have a significant toxic effect on eukaryotic animal cells. We assume that the coating developed by our team may be interesting for application in the medicine and the food industry for long-term antibacterial surface treatment.

## 2. Materials and Methods

### 2.1. Experimental Plant for Production of Nanosized Amorphous Carbon

**Figure 1:**
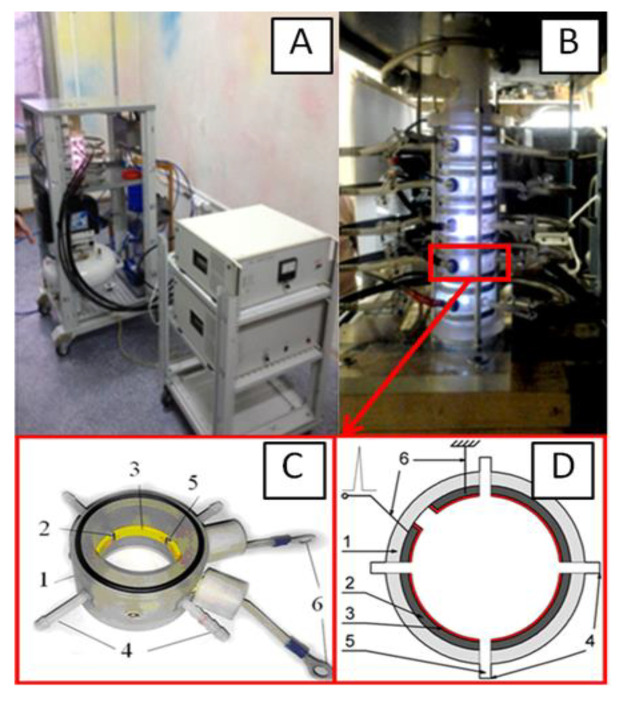
Plant for production of nanosized amorphous carbon: (**A**) General view of the power section; (**B**) Plant reactor in operation; (**C**) Model of one of the reactor blocks; (**D**) Diagram of one of the reactor blocks. 1—dielectric chamber; 2—electrodes; 3—electrically insulating material; 4—branch pipes for air injection into the hole; 5 and 6—terminals for voltage supply.

Experiments to obtain a colloidal solution of nanosized amorphous carbon were carried out on the original plant. The plant consists of a power section, control unit (Figure 1A) and reactor (Figure 1B). The reactor consists of several connected blocks assembled into a column. Each block consists of a dielectric chamber, electrodes, electrically insulating material and argon injector (Figure 1C,D).

**Figure 2:**
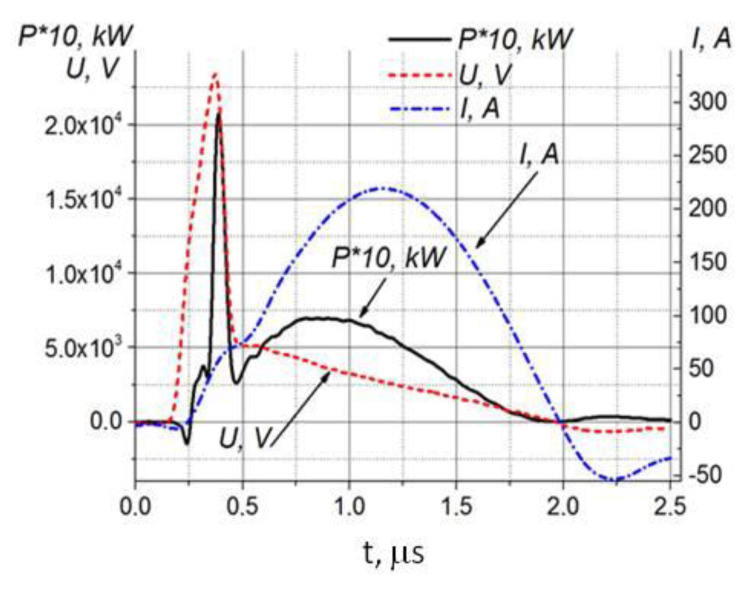
Typical oscillograms of current, voltage and released power when a high-voltage pulse is applied to multi-spark ring plasma arresters of the plant.

Power source parameters: storage capacitor energy W ≤ 1.6 J; voltage U ≤ 20 kV; current I ≤ 300 A; pulse frequency f ≤ 100 Hz. The chamber is filled with 95% ethyl alcohol (Kristal, Moscow, Russia). The consumption of gas (Ar) is 2 L/min. The algorithm for generating nanoparticles is as follows. When a high-voltage pulse is applied to the multi-electrode ring, spark channels are formed in the interelectrode gaps, characterized by the following parameters: temperature of heavy particles Tg ≈ 4000 to 5000 K, electron temperature Te ≈ 1.0 to 1.5 eV, concentration of charged particles ne ≈ (2–3) 10^17^ cm^3^. The spark channel passes through an organic solvent (ethanol), as a result of which, carbon is condensed by alcohol, forming amorphous carbon nanoparticles. Stable colloids were prepared under the same conditions; the processing time was 60 min. Typical oscillograms of current, voltage and released power when a high-voltage pulse is applied to multi-spark ring plasma arresters of the plant are shown in Figure 2.

### 2.2. Producing a Coating of Nanosized Amorphous Carbon

Colloidal solutions of nanosized carbon particles were applied using a spray gun to the surface of the glass slides until completely darkened. The slides were placed in the chamber until the colloid on the surface was completely dry. Colloid drying does not require placing the sample in a vacuum or any additional action. After the evaporation of the liquid component (ethanol), nanosized carbon coating appears on the glass surface. The coating is extremely difficult to separate from the substrate. Mechanical treatment is not able to remove coating without damaging the glass.

### 2.3. Physical and Chemical Methods for Studying Nanosized Amorphous Carbon

Average size and zeta potential were measured using Zeta Sizer Nano ZS device (Malvern, Great Britain). A He-Ne laser with a wavelength of 633 nm was used as a light source. The scattering angle was 173°. The main procedures were described in detail earlier [21]. The coating formed by nanosized amorphous carbon was studied using a JEM-2100 transmission electron microscope in combination with a JED-2300 energy dispersive X-ray spectrometer. The coating was also examined using MIM-207 modulation interference microscope. The Raman spectra of the nanocomposites were recorded with U1000 spectrometer. The cluster sizes were estimated according to [22]. The resulting colloids were investigated by gas chromatography. Analysis of the product generated in organic solvents after their preparation was performed using Chromatec-Crystal 5000.2 gas chromatograph with a flame-ionization detector. The capillary column used was Agilent DB-FFAP, 50 m × 0.32 mm × 0.5 μm. The processing algorithm was previously described [23].

### 2.4. Method for Assessing Bactericidal Activity

Cultivation of bacteria was carried out in an LB medium on microscope slides coated or not coated with nanosized carbon at 37 °C for 24 h. A pseudo-control sample, on which ethanol was evaporated, was also tested for bacterial colonization. The tests used a bacterial suspension consisting of *Escherichia coli* (gram-negative) and *Staphylococcus aureus* (gram-positive) organisms. After incubating the slides for the required period, the film side of each slide was washed and stained using crystal violet and examined by a microscope at ×1000 magnification. Experimental details were previously described [24].

### 2.5. Cell Culture

**Figure 3:**
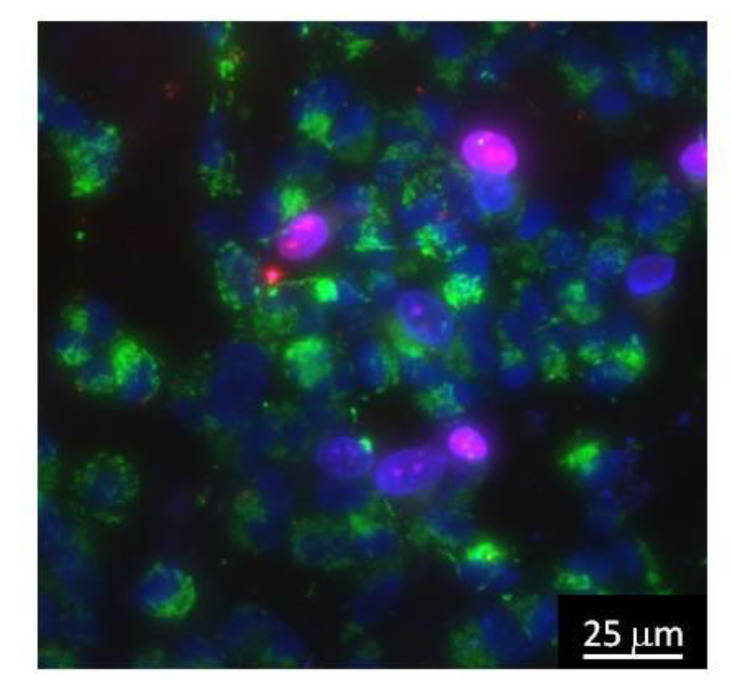
A typical micrograph of cell culture. The mitochondria of the cells are colored green; they may be used to estimate cell size. Normal cell nuclei are colored blue. Nuclei of non-viable cells are colored purple.

The cytocompatibily study of the alloys was carried out using standard in vitro test systems. SH-SY5Y cells were used as standard cell models. Cells were cultured on plates with or without nanosized carbon. To determine the number of living and dead cells, they were stained with fluorescent dyes, Hoechst 33342 (Sigma, St. Louis, MO, USA)—2 μg/mL and propidium iodide (Sigma, St. Louis, MO, USA)—2 μg/mL. Hoechst 33342 dye stains all cells (living and dead) and propidium iodide dye quickly penetrates only cells with damaged membranes (dead). Additionally, for contrast, cells were stained with the mitochondrial dye, MitoTracker Green FM (Thermo Fisher Scientific, Waltham, MA, USA)—2 μg/mL. For analysis, at least 500 cells on the surface of each sample were counted [25]. A typical micrograph of cell culture is shown in Figure 3.

The number of cells in a state of mitosis was determined by fluorescence microscopy using intravital staining with Hoechst 33342 fluorescent dye (Sigma, St. Louis, MO, USA). Mitotic cells were detected by the chromatin distribution characteristic of prophase (P), metaphase (M), anaphase (A) and telophase (T). For analysis, at least 500 cells on the surface of each sample were counted. The mitotic index (MI) was calculated using the equation MI = (P + M + A + T)/N × 100%, where (P + M + A + T) is the number of cells at the prophase, metaphase, anaphase, and telophase stages, and N is the total number of cells analyzed [26].

## 3. Results

**Figure 4:**
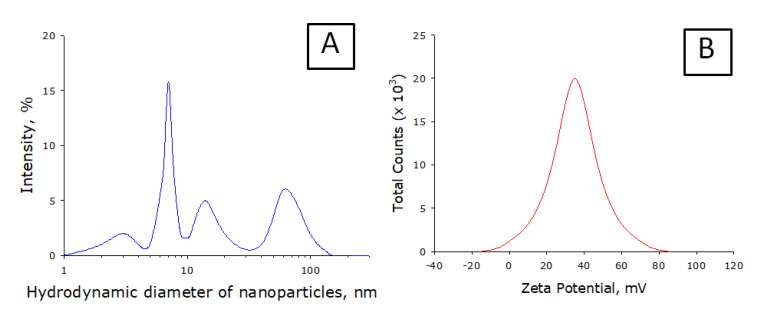
Properties of nanosized carbon particles: (**A**) Nanoparticle size distribution; (**B**) Zeta potential profile of nanoparticles.

It is shown that when the specific energy input to ethanol is exceeded by more than 10 J/cm^3^, a stable colloidal solution of amorphous carbon nanoparticles is formed. Specific energy input was defined as electrical energy in the discharge against the unit of ethanol volume. The size distribution of nanoparticles generated by the installation does not change significantly with an increase in the voltage applied to the installation electrodes. A typical nanoparticle size distribution is shown in Figure 4A. The formation of nanoparticles of different sizes occurs. The smallest nanoparticles have a hydrodynamic diameter of 2 to 4 nm. Additionally, in the colloidal solution, nanoparticles with typical sizes of 7, 15, and 67 nm are observed. The peak corresponding to nanoparticles with an average hydrodynamic diameter of 7 nm has the highest intensity. It is shown that the size distribution of nanoparticles contains several fractions. Larger fractions can be aggregates of smaller nanoparticles. It is known that, upon aggregation, the size distribution of nanoparticles changes significantly over time. When storing a colloidal solution of nanosized carbon particles for 3 months, there is no significant change in the size distribution of nanoparticles. No additional aggregation was noted. With a high degree of probability, it can be argued that the distribution contains only true nanoparticles, no aggregates. Such stability of nanoparticles colloid should be due to a high zeta potential. Figure 4B shows the zeta potential profile of nanoparticles. It is shown that the width of the zeta potential distribution (order of 100 mV) is −15 to 85 mV. The distribution half-width is 25 to 55 mV. The function extremum is 34.3 mV. Such a zeta potential profile allows this assuming that only carbon nanoparticles are present in the solution.

It was established by gas chromatography that during the plasma treatment of ethanol, in addition to nanosized carbon, a large number of chemical compounds are formed. Acetaldehyde and butanol-1 have the highest yield. Ethyl acetate, propanol-2 and methanol are formed in smaller amounts by one order of magnitude. Acetone, propanol-1, butanol-2, butanone-2 and isobutyl ether are found in trace amounts. The heating of the colloidal solution up to a temperature close to ethanol boiling point and the subsequent cooling does not significantly change its properties.

**Figure 5:**
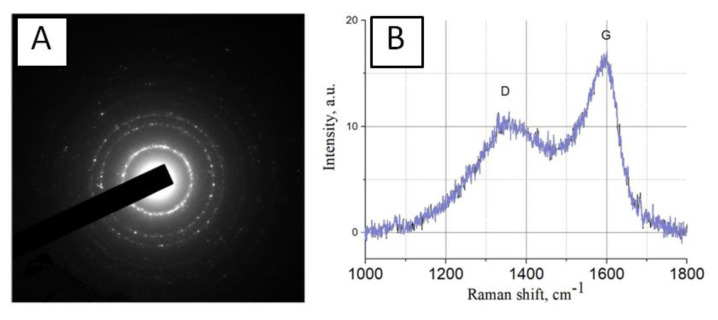
Properties of nanosized carbon particles: (**A**) Two-dimensional X-ray pattern obtained for carbon nanoparticles sample; (**B**) Raman spectrum of carbon nanoparticles sample.

The two-dimensional X-ray pattern (Figure 5A) obtained for the carbon nanoparticles sample shows that the size of one cluster is about 2 nm. The analysis of the elemental composition showed that the composition of carbon nanoparticles contains atomized metals of the electrodes. The total metal content is not more than 2% by weight. The main elements are iron (71%), chromium (18%), nickel (10%), silicon (0.7%) and copper (0.3%). Raman spectroscopy confirms the findings on the size of individual nanoparticles. Figure 5B shows peaks D (1350 cm^−1^) and G (1595 cm^−1^) in the Raman spectrum of carbon nanoparticles sample. This means that the main part of the sample is disordered carbon in the form of graphite nanoparticles. The size of the graphite clusters may be estimated from the ratio between the intensities of the components in D and G spectrum peaks. The calculated average diameter of one cluster is about 1 to 2 nm.

**Figure 6:**
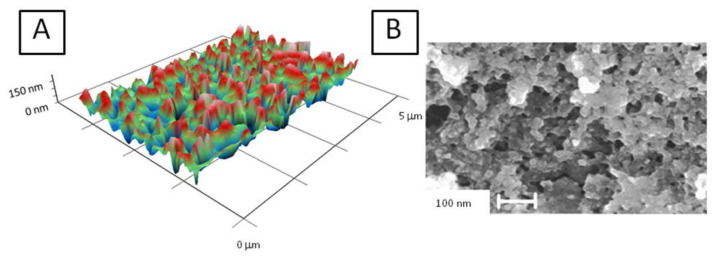
Micrographs of the surfaces formed by the nanosized carbon coating: (**A**) Image obtained using a modulation interference microscope; (**B**) TEM micrograph.

It is obvious that the structure of the sample may change significantly in the colloidal solution and on the surface after drying. To establish the morphology of nanosized carbon coatings, the resulting colloidal solution was applied to flat surfaces and dried. The resulting nanosized carbon coating was tested using modulation interference microscopy (Figure 6A) and TEM (Figure 6B).

**Figure 7:**
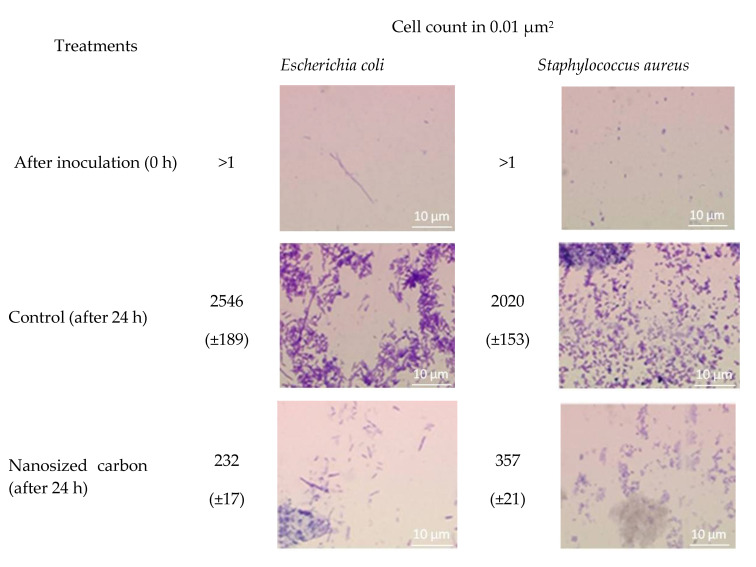
Effect of nanosized carbon coating on the development of Escherichia coli and Staphylococcus aureus. Incubation time is 24 h. For nanosized carbon, the most abundant micrographs are presented. Data are presented as mean values and standard errors.

The effect of nanosized carbon coating on the development of Escherichia coli and Staphylococcus aureus was studied (Figure 7). It was shown that both bacilli (E. coli) and cocci (S. aureus) divide and attach to the control surface well. Growth of Escherichia coli and Staphylococcus aureus on nanosized carbon coatings shows significant inhibition. For E. coli, a decrease in growth rate by more than one order of magnitude is observed. For S. aureus, growth rate decreases by almost six times.

**Figure 8:**
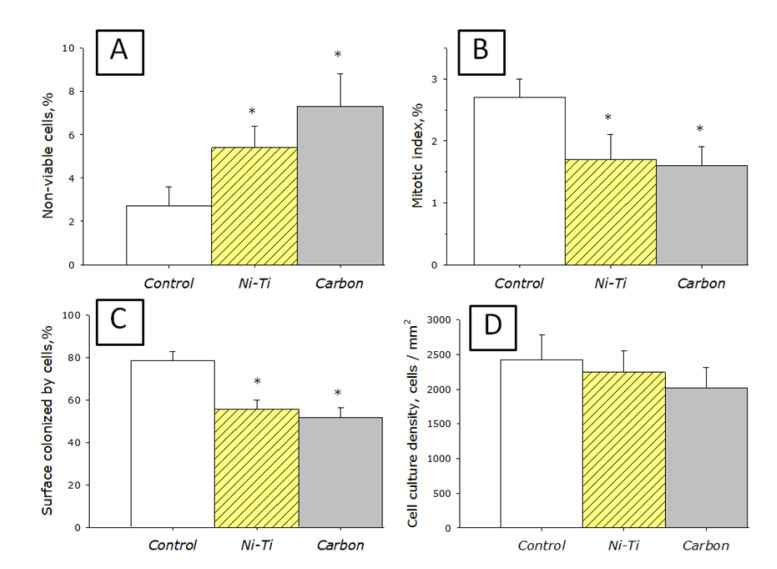
Effect of nanosized carbon coating on the main characteristics of cell culture growth and development: (**A**) Effect of nanosized carbon coating on cell viability; (**B**) Effect of nanosized carbon coating on the mitotic index of the cell population; (**C**) Effect of nanosized carbon coating on the rate of available area colonization; (**D**) Effect of nanosized carbon coating on cell culture density. Control—glass slide. Ni-Ti—nitinol medical alloy for the implant manufacture. Carbon—samples with nanosized carbon coating. *—statistically significant differences from the control (*p* < 0.05).

The effect of a nanosized carbon coating on the growth and development of cell cultures was studied (Figure 8). A glass slide and a titanium nickelide medical alloy (Ni-Ti, nitinol) were used as controls. The effect of nanosized carbon coating on the viability of cell cultures was investigated (Figure 8A). The number of non-viable cells grown on a glass slide did not exceed 3%. When cells were grown on nitinol, the number of non-viable cells was about 5%. When cells were grown on nanosized carbon coating, their number was about 7%. It should be noted that the data on the viability of cells grown on medical alloy and nanosized carbon coating did not differ statistically. However, when nitinol or nanosized carbon coatings are used as a substrate, the number of non-viable cells increases almost two times. In general, all studied materials do not have a significant toxic effect on cell cultures.

To analyze the ability of cells to divide, the mitotic index of cells in the logarithmic growth phase was determined (Figure 8B). Cells in the process of division were identified by the distribution of chromatin typical for mitosis (prophase, metaphase, anaphase, and telophase). It was found that the mitotic index of cells growing on the glass slide surface for the SH-SY5Y culture is about 2.5%. When using nitinol plates or nanosized carbon coating as a substrate, the mitotic index is about 1.5%. Thus, the mitotic index of cell cultures growing on the surface of nanosized carbon coating corresponds to the mitotic index of cells growing on the nitinol medical alloy. At the same time, the mitotic index of cells on the glass slide is one third higher.

After 72 h of cultivation, a morphological analysis of the cells on the surface of the materials was carried out. It was found that the glass slide surface is more suitable for cell attachment and spreading than the surface of nanosized carbon coating or nitinol (Figure 8C). It was found that for 72 h on all investigated surfaces (glass slide, nitinol, nanosized carbon coating), cells do not form a continuous monolayer. Although in some parts of the culture there are some monolayer elements. On the glass slide surface, cells occupy about 80% of the surface available for growth. On the nitinol surface and nanosized carbon coating, cells occupy about half of the available area.

It was shown that when cells are cultured on the glass slide, nitinol and nanosized carbon coating, the density of the cell culture is slightly more than 2000 cells per mm^2^ in average (Figure 8D). The highest cell density is observed on the glass slide; slightly less density is typical for nitinol and nanosized carbon coating. Thus, when cells are cultured on nanosized carbon coating, the same cell density is achieved as when cultured on the glass slide or nitinol medical alloy.

## 4. Discussion

Currently, there are several competing methods for obtaining nanosized carbon (electric arc graphite spraying [27], laser evaporation of graphite [28], chemical deposition from vapor [29], etc.). We have proposed a relatively simple method for producing nanoparticles, the main advantage of which is its low cost (raw material is ethanol, medium electricity consumption) and high speed (2 L of colloid per minute from one plant). Another significant advantage of the method is the colloidal form. In principle, a colloid containing nanosized carbon particles does not require additional processing before being applied to surfaces. This is probably due to the chemical conversion of some ethanol part. We have shown that acetaldehyde and butanol-1 have the highest yield. Obviously, with such products, the main reaction in plasma is the abstraction of a hydrogen atom from an alcohol molecule. Probably, this reaction is universal, since earlier similar results were obtained during laser breakdown of ethanol containing gold nanoparticles [22]. Ethyl acetate, propanol-2 and methanol are formed in much smaller quantities. This means that the reaction of C-C bond breaking in plasma is less preferable. Any remaining compounds may be the products of these two basic reactions. Moreover, these reactions are likely to take place at the plasma boundary and do not play an important role in nanoparticles generation. On the other hand, the obtained new organic compounds may significantly increase the adhesive properties and solubility of nanoparticles [30].

The generation of nanoparticles occurs directly in the plasma. We have shown that traces of metals are found in the sample. However, the zeta potential profile of the nanoparticles has one peak and is symmetric (Figure 4B). This suggests that metal ions may be implanted from nanoparticles (there are not two types of nanoparticles in the colloid). It is known that metal ions can be toxic to living systems. This is especially true for metal ions of variable valence. The complex size distribution (Figure 4A) is associated only with carbon nanoparticles. The X-ray pattern (Figure 5A) and the Raman spectrum (Figure 5B) show that one type of nanosized carbon with a size of about 2 nm is most likely formed. We believe that the presence of different nanoparticle sizes is due to the aggregation of 2 nm nanoparticles. Recalculation of the light scattering intensity into the number of particles using the equation [31] shows that there are almost 107 nanoparticles with a size of 2 to 3 nm per one 67 nm nanoparticle. There are about 7000 small nanoparticles with a size of 2 to 3 nm per one 15 nm nanoparticle. There are about 200 small nanoparticles per one 7 nm nanoparticle. Microscopy results also indicate that there is only one type of nanoparticle and its aggregates in solution (Figure 6). Grains of nanosized carbon coating are irregular, with different sizes and shapes. Perhaps such irregularity is one of the reasons for the bacteriostatic effect [32] observed when growing bacteria on nanosized carbon coatings (Figure 7). In principle, the antimicrobial activity of coatings may be explained by several reasons. Generated nanosized carbon coatings are highly hydrophobic. It is known that the strong hydrophobicity of coatings may cause membrane changes in a number of microorganisms [33]. It is believed that the sp3/sp2 ratio plays an important role in the biological activity of nanosized carbon materials [34]. Our coating has an sp3/sp2 ratio of about 6. Part of the antibacterial effect may be associated with the generation of reactive oxygen species on the surface of carbon coatings [35]. Interestingly, while having pronounced antibacterial activity, nanosized carbon coatings have almost no effect on the development of eukaryotic cells. It was shown that cells growing on nitinol medical alloy and nanosized carbon coating have similar growth and development rates (Figure 8). Of course, nitinol is not the most modern and advanced medical alloy [36], but it is still widely used for the manufacture of implants.

## 5. Conclusions

Thus, an electrospark technology has been developed for obtaining a colloidal solution containing nanosized amorphous carbon. The advantages of the technology are its low cost and high performance. The colloidal solution of nanosized carbon is highly stable. The coatings on its basis are nanostructured. They are characterized by high adhesion and hydrophobicity. It was found that the propagation of microorganisms on nanosized carbon coatings is significantly hindered. At the same time, eukaryotic animal cells grow and develop on nanosized carbon coatings, as well as on the nitinol medical alloy. The use of a colloidal solution as available, cheap and non-toxic nanomaterial for the creation of antibacterial coatings to prevent biofilm formation seems to be very promising for modern medicine, pharmaceutical and food industries
